# Dental Medicine

**Published:** 1867-10

**Authors:** 


*Dental Medicine*—We are much indebted to Dr. S. S. White for some very fine preparations prepared especially for the dental profession. They consist of Dr. Benj. Richardson's Styptic Colloid, a notice of which appeared in the July No. of the *Am. Journal of Dental Science;* also compounds of Carbolic acid and Glycerin, and of Iodine and Glycerin.

The two last preparations we have for a long time, been successfully using for fetid discharges and cases of chronic abscess.

We have also received from Dr. White an illustrated Dental Catalogue, the finest work of the kind ever issued.

